# Global Cross-Sectional Study Evaluating the Attitudes towards a COVID-19 Vaccine in Pregnant and Postpartum Women

**DOI:** 10.3390/vaccines11020390

**Published:** 2023-02-08

**Authors:** Natalie D. Hernandez, Sally Pairman, Alan C. Fisher, Ru-fong J. Cheng, Shirley Sylvester

**Affiliations:** 1 Department of Community Health and Preventive Medicine, Morehouse School of Medicine, Atlanta, GA 30310, USA; 2 International Confederation of Midwives, 2514 AE The Hague, The Netherlands; 3 Alan C Fisher, LLC, West Orange, NJ 07052, USA; 4 Office of the Chief Medical Officer, Johnson & Johnson, New Brunswick, NJ 08901, USA

**Keywords:** COVID-19, vaccine acceptance, vaccination, pregnancy, postpartum, global

## Abstract

Pregnant and postpartum women have an increased risk of severe complications from COVID-19. Many clinical guidelines recommend vaccination of these populations, and it is therefore critical to understand their attitudes toward COVID-19 vaccines. We conducted a cross-sectional online survey in November 2020 of currently pregnant and ≤1-year postpartum women in Brazil, India, the United Kingdom (UK), and the United States (US) that assessed their openness to COVID-19 vaccines and reasons for vaccine hesitancy. Logistic regression analyses were conducted to evaluate openness to receiving a vaccine. Out of 2010 respondents, 67% were open to receiving a COVID-19 vaccine themselves. Among pregnant and postpartum participants, 72% and 57% were willing to receive a vaccine, respectively. Vaccine openness varied significantly by country: India (87%), Brazil (71%), UK (59%), and US (52%). Across all participants, among the 33% who were unsure/not open to receiving a COVID-19 vaccine, the most common reason cited was safety/side effect concerns (51%). Participants were similarly open to their children/other family members receiving a COVID-19 vaccine. Presence of a comorbidity, a positive COVID-19 test result, and pregnancy were all significantly associated with positive vaccine acceptance. Targeted outreach to address pregnant and postpartum women’s concerns about the COVID-19 vaccine is needed.

## 1. Introduction

The novel coronavirus disease 2019 (COVID-19) pandemic has profoundly impacted communities around the world. Compared to nonpregnant women of reproductive age, pregnant and recently pregnant women with COVID-19 have a higher risk of severe complications, including intensive care unit admission and need for invasive ventilation [1] (We recognize that there are more inclusive terms for individuals who give birth [e.g., birthing people]. However, the existing literature and legal system largely use the term “pregnant women” and we enrolled pregnant women in our study. Therefore, the same language is throughout our manuscript.). Pregnant women with COVID-19 also face an increased risk of death and preterm birth, and their infants are at an increased risk of neonatal intensive care unit admission and stillbirth [1].

Vaccination against SARS-CoV-2 has demonstrated effectiveness in reducing disease severity and it is considered an important public health intervention to control the pandemic. Several highly effective COVID-19 vaccines have been authorized or approved for use globally. However, pregnant and lactating women were excluded from preauthorization clinical trials and only limited human data on safety during pregnancy and lactation were available at the time of the emergency authorization. Safety and effectiveness data from real world use of the vaccines in pregnant and lactating women are accumulating [2,3,4,5,6,7,8]. Similar to the general population, available data indicate that the vaccines are safe and effective in pregnancy [9,10,11,12]. Available data on fetal and neonatal safety are also reassuring, and maternal vaccination has been shown to confer some short-term immunity to the infant [9,10,12,13,14].

Various clinical guidelines support COVID-19 vaccination during pregnancy and breastfeeding, including those from the United States (US) Centers for Disease Control and Prevention (CDC), the American College of Obstetricians and Gynecologists, and the Society for Maternal–Fetal Medicine in the US [15]. As more data have become available about the risks of COVID-19 in pregnancy and vaccine safety and efficacy in pregnancy, these professional organizations now strongly recommend getting the vaccine during pregnancy [16,17]. Similarly, the World Health Organization has stated that “the benefits of vaccination during pregnancy outweigh potential risks whenever there is ongoing or anticipated community transmission of the virus” [18]. According to data from the Johns Hopkins University COVID-19 Maternal Immunization Tracker, the vast majority of countries/territories around the world currently recommend or permit the COVID-19 vaccine during pregnancy (120 recommend a COVID-19 vaccine for some or all pregnant women, 61 permit the vaccine, 9 permit it with qualifications, 12 do not recommend it, and 22 have no position on the matter) [19].

However, despite the recommendations from national and global organizations to vaccinate pregnant women against COVID-19, vaccine hesitancy, among other factors, may result in low uptake. In several meta-analyses, which used partially overlapping studies, COVID-19 vaccine acceptance among pregnant/postpartum women ranged from 47 to 62% [20,21,22,23,24,25]. Well into 2021, actual vaccination rates in pregnant women were lower. A meta-analysis of 703,004 pregnant women (eleven studies with data through October 2021) found that overall 28% of pregnant women in five countries (Israel, Japan, Scotland, United Kingdom [UK], US), were vaccinated against COVID-19 [26].

An understanding of the attitudes of pregnant and lactating individuals towards the COVID-19 vaccine and underlying reasons for vaccine hesitancy may lead to improved vaccination rates that help protect these high-risk individuals from complications of COVID-19. As mothers make important health care decisions about their families, it is also important to understand their openness to COVID-19 vaccination of their families. To that end, we conducted a cross-sectional, exploratory study in four countries (US, UK, Brazil, and India) to identify pregnant and postpartum women’s attitudes towards COVID-19 vaccines, particularly regarding their willingness to receive a potential vaccine, openness to a family member receiving a vaccine, and reasons for vaccine hesitancy.

## 2. Methods

### 2.1. Ethics Approval

The survey protocol was approved by Solutions IRB. No personally identifiable data were collected. All participants provided informed consent before participating in the survey and agreed to take part in online research and to privacy policies.

### 2.2. Survey Participants

Women were eligible to participate in the survey if they were aged 18–59 years (age 59 was chosen to be inclusive of all potential participants especially those achieving pregnancy with assistive reproductive techniques); currently pregnant or up to one year postpartum; resided in Brazil, India, the UK, or the US; and were able to read and understand the questionnaire. These four countries were chosen for having differing levels of medicalization of births, politicization of COVID-19, and COVID-19 incidence, as well as alignment with where COVID-19 vaccines were being studied.

### 2.3. Recruitment

The survey was conducted between 11 November and 16 November 2020, before COVID-19 vaccines were widely available. Recruitment stopped when approximately N = 2000 (N = 500 per country) participants were screened, had provided consent, and completed the survey. A power calculation was conducted and 500 participants per country was deemed adequate to detect medium effect sizes on key measures between countries.

All recruitment and data collection used an online research panel. Panelists were obtained from proprietary customer loyalty partnerships, open recruitment traditional online panels, including people recruited from mobile app panels, and integrated partnerships with online communities, publishers, and social networks. This multi-sourced recruitment model increases reach and capacity and improves consistency. Panelists were matched with available surveys using a platform that employs multiple points of randomization and received notifications when surveys were available. Panelists who completed the study received an incentive, which varied slightly across panelists and in the different countries of study. Panelists were offered points in an amount and “currency” that was relevant to their membership. For example, those participants who were members of an air miles subpanel received points in air miles.

The research was structured in a way that reduced response bias tendencies such as social desirability bias (the tendency to select responses participants believe will make them look good to others) and position bias (the tendency to select responses listed earlier in a set of options) that can occur with online surveys. As part of the informed consent process, research participants knew that their identity and personal information were not shared with the research team, which helped reduce response bias tendencies to answer in the direction of social desirability. In addition, where applicable, response order was randomized to reduce the impact of position bias.

### 2.4. Questionnaire

This exploratory cross-sectional study was conducted using a knowledge, attitude, and practice online survey. Participants completed the survey in their local language using any device with internet access such as their personal computers or smartphones.

The survey required about 20 min to complete and covered: demographic information (race, ethnicity, and age); pregnancy status, information, and history (e.g., first or not first time being pregnant/giving birth, vaginal vs. C-section, etc.); current health and comorbidities; and experiences/behaviors of interest (healthcare experience, healthcare-seeking behaviors, mental health and well-being, and attitudes toward a COVID-19 vaccine).

This report focuses on summarizing some of the key demographic characteristics of the participants and on the questions assessing vaccine acceptance: (1) Once a COVID-19 vaccine is available, how open would you be to taking a vaccine to prevent COVID-19 during pregnancy or postpartum? (2) For those who responded “Neutral/Unsure”, “Not Very Open”, or “Not Open at All” to Question 1: Why are you unsure or not open to taking a COVID-19 vaccine once it is available? (3) Once a COVID-19 vaccine is available, how likely are you to allow your child/children or other family member(s) to take a vaccine to prevent COVID-19?

For questions 1 and 3, participants were asked to select “Open”, “Very Open”, “Neutral/Unsure”, “Not Very Open”, or “Not Open at All.” For question 2, respondents were asked to select “Lack of compelling information on benefits”, “Safety and side effect(s) concerns”, “Mistrust of health and/or political authorities”, “Conspiracy theories worry me”, or “I do not believe vaccinations (general) provide any benefit.”

### 2.5. Statistical Analysis

Logistic regression analyses were conducted to evaluate openness to receiving a vaccine. Responses of “Open” and “Very Open” were categorized as “Open” and “Neutral/Unsure”, “Not Very Open”, and “Not Open at All” were categorized as “Neutral/Not Open.” To determine the potential prognostic variables for each country, both univariate and multivariate logistic regression analyses were conducted. Potential prognostic variables included age cohort, pregnancy status, COVID-19 status, and any comorbid condition. Odds ratios and the corresponding 95% confidence intervals were calculated for each prognostic variable, with odds ratios greater than one indicating that the respondents in the reference group were more open to vaccine acceptability. Statistical analyses were conducted using SAS version 9.4.

## 3. Results

### 3.1. Baseline Characteristics of Participants

The baseline characteristics of the respondents are summarized in Table 1. Among the 2010 respondents, 69.2% were pregnant, 54.8% were 30–39 years of age, and 46.4% had a comorbidity. Among the 1257 respondents who had ever taken a viral COVID-19 test (which identifies a current infection), 28.4% tested positive.

### 3.2. Openness to Self Receiving a COVID-19 Vaccine

**Figure 1:**
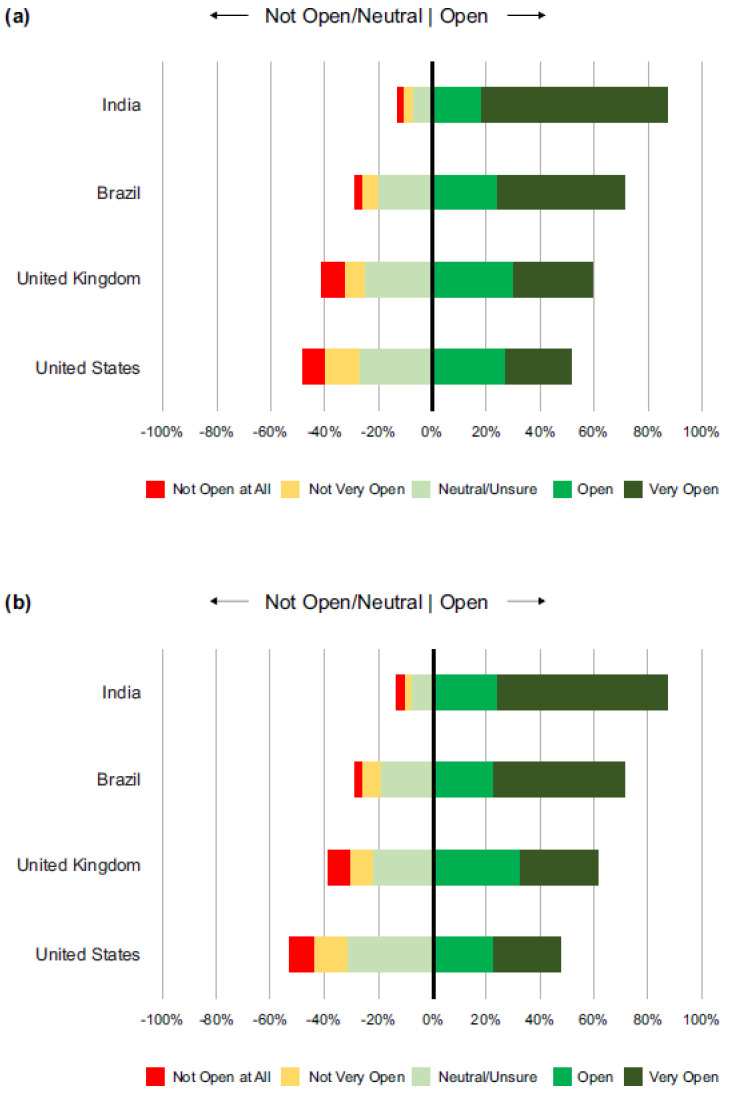
Openness for self (**a**) and children/other family members (**b**) to receive a COVID-19 vaccine, by country.

Among the 2010 respondents, 67.4% were “Very Open” or “Open”, 19.5% were “Neutral/Unsure”, and 13.1% were “Not Very Open” or “Not Open at All” to receiving a COVID-19 vaccine. Among the 69% of participants who were pregnant at the time of the survey, 72.2% were “Very Open” or “Open”, 16.6% were “Neutral/Unsure”, and 11.2% were “Not Very Open” or “Not Open at All” to receiving a COVID-19 vaccine. Among the 31% of participants who were postpartum, 56.8% were “Very Open” or “Open”, 26.0% were “Neutral/Unsure”, and 17.3% were “Not Very Open” or “Not Open at All” to receiving a COVID-19 vaccine. As summarized in Figure 1a, vaccine openness (responses of “Very Open” or “Open”) varied widely across the respondents in the four countries: 51.8% in the US, 59.2% in the UK, 71.2% in Brazil, and 87.1% in India.

### 3.3. Reasons for Vaccine Hesitancy

**Figure 2:**
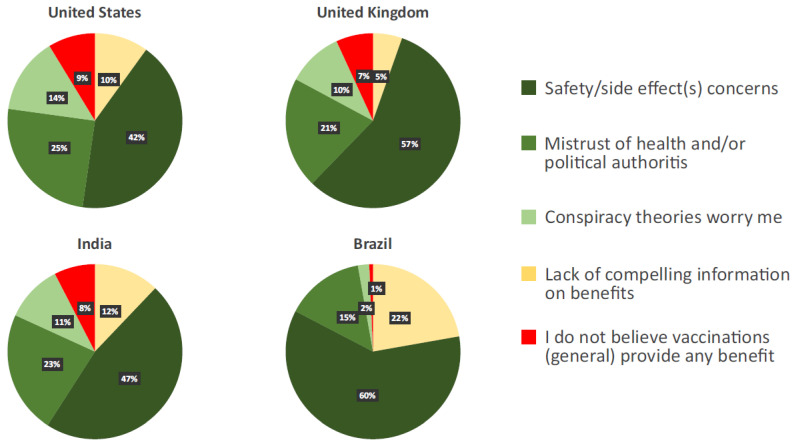
Reasons for hesitancy to receive a COVID-19 vaccine among respondents who selected Neutral/Not Open.

Across all countries, of the 32.6% of participants who responded “Neutral/Unsure”, “Not Very Open”, or “Not Open at All” to receiving a vaccine, the most common reason cited was “Safety/side effect concerns” (51.3%), followed by “Mistrust of health/political authorities” (21.1%), “Lack of compelling information on benefits” (11.5%), “Conspiracy theories worry me” (9.9%), and “I do not believe vaccinations (general) provide any benefit” (6.3%). “Safety/side effect concerns” was also the most common reason cited in each of the four countries (Figure 2).

### 3.4. Openness to Children/Other Family Members Receiving a COVID-19 Vaccine

Responses were very similar to the self-receiving the vaccine question. Among the 2010 respondents, 67.0% were “Very Open” or “Open”, 19.5% were “Neutral/Unsure”, and 13.6% were “Not Very Open” or “Not Open at All” to taking a COVID-19 vaccine. Among the 69% of participants who were pregnant at the time of the survey, 71.7% were “Very Open” or “Open”, 17.3% were “Neutral/Unsure”, and 10.9% were “Not Very Open” or “Not Open at All” to receiving a COVID-19 vaccine. Among the 31% of participants who were postpartum, 56.3% were “Very Open” or “Open”, 24.2% were “Neutral/Unsure”, and 19.5% were “Not Very Open” or “Not Open at All” to receiving a COVID-19 vaccine. Vaccine openness by country is shown in Figure 1b.

### 3.5. Predictors of Vaccine Acceptance

**Figure 3:**
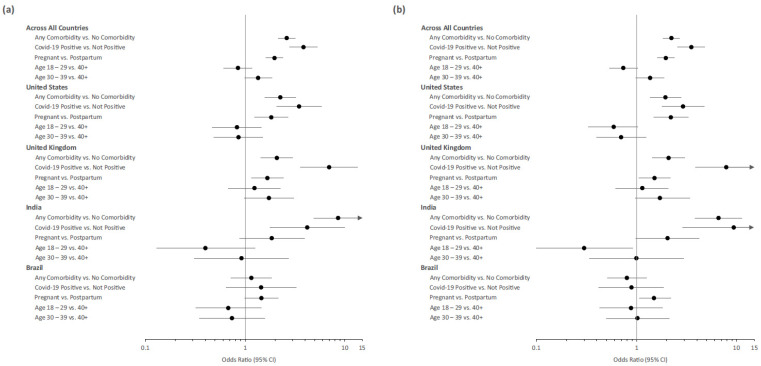
Univariate (unadjusted) Odds Ratio (95% CI) by country and across countries for potential prognostic factors for openness to oneself (**a**) and one’s children/other family members (**b**) receiving a COVID-19 vaccine (vs. Neutral/Unsure and Not Open) among pregnant and postpartum women. Odds ratios greater than one indicate that the respondents in the comparison group were more open than the reference group to vaccine acceptability.

Univariate analyses across the four countries for potential prognostic factors for (1) openness to receiving a COVID-19 vaccine oneself and (2) openness to children/other family members receiving a COVID-19 vaccine are shown in Figure 3, Supplementary Tables S1 and S2. Presence of a comorbidity, a positive COVID-19 test result, and pregnancy were all significantly associated with positive vaccine acceptance.

The associations between openness to receiving a COVID-19 vaccine oneself and potential predictors are summarized by country in Figure 3 and Supplementary Table S1. Among respondents in the US and the UK, being pregnant, having a positive COVID-19 status, and having a comorbidity were significantly associated with positive vaccine acceptance. Among respondents in India, positive COVID-19 status and comorbidity were significantly associated with positive vaccine acceptance. The odds ratio not showing statistical significance for pregnancy status being a predictor of vaccine acceptance was likely due to the imbalance of pregnant and postpartum women surveyed in India, with only 49 women postpartum. Although similar trends were observed among respondents in Brazil as observed in the other three countries, none of the odds ratios in Brazil were statistically significant.

Similar results were found when participants were asked about their willingness for their children/other family members to receive a COVID-19 vaccine (Figure 3 and Supplementary Table S2). Among respondents in the US and the UK, being pregnant, positive COVID-19 status, and comorbidity were significantly associated with positive vaccine acceptance. Among respondents in India, positive COVID-19 status and comorbidity were significantly associated with positive vaccine acceptance. Although similar trends were observed among respondents in Brazil as observed in the other three countries, only being pregnant was statistically significantly associated with positive vaccine acceptance in Brazil.

There does not appear to be a significant association between age group and vaccine acceptance within any of the countries or across all countries for either question, except for being age 18–29 in India, which was significantly associated with negative vaccine acceptance for the willingness for children/other family members to receive a COVID-19 vaccine question.

As expected, the adjusted odds ratios estimated from the multivariate analyses (Supplementary Tables S3 and S4) were directionally closer to unity than the results observed with the univariate analyses.

## 4. Discussion

In this cross-sectional, exploratory study to identify pregnant and postpartum women’s attitudes towards COVID-19 vaccines, we found that overall vaccine openness was 67%, acceptance among pregnant women was 72%, and acceptance among postpartum women was 57%. Vaccine openness varied widely by country, ranging from 52% to 87%. The most common reason for vaccine hesitancy was “safety/side effect concerns.” Presence of a comorbidity, a positive COVID-19 test result, and pregnancy were all significantly associated with positive vaccine acceptance. Nearly identical results were obtained when participants were asked about receiving a vaccination themselves and vaccinating their children or other family members (67.4% and 67.0% openness, respectively).

Our results are consistent with findings from a recent global survey of COVID-19 vaccine attitudes among pregnant/postpartum women from 16 countries by Skjefte et al., which was conducted in the same time frame as our study and included the four countries we surveyed [27]. In their study, as in ours, of the four countries, the US had the lowest rate of vaccine acceptance, followed by the UK, then Brazil, and then India. The vaccine acceptance percentages by country were roughly similar in both studies. In addition, the same vaccine acceptance rate (67%) was observed across all women (pregnant and non-pregnant mothers in Skjefte et al. and pregnant and postpartum women in our study). Further, sixty-nine percent of pregnant and non-pregnant women intended to vaccinate their children in the Skjefte study, compared to 67% in our study. Of note, and perhaps surprisingly, in both studies, the same acceptance rate was observed when participants were asked about vaccinating themselves or their children/other family members.

A systematic review of COVID-19 vaccine acceptance among pregnant women also noted higher levels of vaccine acceptance in East and Southeast Asia (including India) as well as some South American countries (including Brazil) relative to most of Europe and North America [28]. There are many potential reasons for such geographical variation in COVID-19 vaccine acceptance among pregnant and postpartum women. One potential reason is that the pandemic has followed distinct timelines in different countries. Residents of countries who experienced their first wave peaks of cases earlier (US and UK) may have been less concerned about vaccination than those in countries whose first peak occurred later (India and Brazil), for whom the threat of COVID-19 felt more immediate [29]. Differences in COVID-19-related recommendations between countries—which range from vaccine restriction in pregnancy to full recommendation without restrictions [19,30,31,32]—may also contribute to variation in vaccine acceptability.

Many other studies examining vaccine attitudes among pregnant/postpartum women have been published [33,34,35,36,37,38,39,40,41,42,43,44], and several meta-analyses/systematic reviews on this topic have also been conducted [10,20,21,22,23,24,25,28]. Among the meta-analyses, which used partially overlapping studies, COVID-19 vaccine acceptance rates among pregnant/postpartum women ranged from 47 to 62% [20,21,22,23,24,25], lower than the 67% observed in the current study. Our survey was conducted in November 2020, towards the end of the first year of the pandemic and before vaccines were widely available. Relative to the other studies conducted on the topic, our survey was conducted relatively early in the pandemic. One meta-analysis on vaccine acceptance in pregnant and breastfeeding women conducted a sub-analysis by study period, finding that acceptance ranged from 60% among studies conducted in 2020, to 42% among studies conducted in the first half of 2021, to 62% among studies conducted in the second half of 2021 [23].

In the US and UK, where current data are available, the rates of actual vaccine uptake are higher than the theoretical willingness to be vaccinated in November 2020 reported here, likely due to a reduction in conflicting and incorrect information surrounding COVID-19 vaccines and the growing body of published research (and subsequent public dissemination of these data and education of pregnant women) demonstrating that COVID-19 vaccinations are safe and effective during pregnancy [9,10,11,12]. For example, according to data from 29 October 2022, 71% of US pregnant women aged 18–49 have received two doses of a COVID-19 vaccine [45], higher than the 52% of US participants in our study who reported that they would be open to receiving a vaccine. According to June 2022 data from the UK, 73% percent of women who gave birth in England had received at least one dose of the vaccine before delivery and 67% had received two or more doses before delivery [46]. In our study, 60% of UK respondents said they were very likely or likely to receive a vaccine. Brazil lags behind the US and UK in terms of COVID-19 vaccine uptake. Calculations based on the number of women giving birth in the country in 2019 indicate that less than 30% of pregnant or postpartum women had received a COVID-19 vaccine in Brazil by early August 2021 [47]. This number rose to 40% by the end of March 2022 [48]. Even fewer pregnant women have been vaccinated in India. In a survey that ended in November 2021, 13% of pregnant women in India were vaccinated against COVID-19 [49]. Notably, of the four countries in our survey, India was the last to grant approval for vaccination of pregnant women (2 July 2021) [50]. Additionally, there are likely differences in vaccine education of pregnant women across the countries studied.

Lower vaccine coverage is not due entirely to vaccine hesitancy; lack of access and other factors also play a role. For example, lack of knowledge of the vaccines, inconsistent and unclear guidelines regarding vaccination in pregnancy, limited vaccine supply, transportation issues to vaccine clinics, poor infrastructure for distributing vaccines, and funding shortages for surveillance are all obstacles in lower- and middle-income countries (LMICs) such as India and Brazil [51]. Even in high-income countries such as the US and the UK, there were vaccine accessibility issues, including difficulties with vaccine scheduling and transportation, particularly among Black and Hispanic populations (who have a higher degree of health disparities compared to non-Hispanic White populations), especially early in the pandemic [52]. While addressing vaccine hesitancy is certainly important, finding solutions to other causes of low vaccine coverage is also critical.

In our study, across all countries, presence of a comorbidity, pregnancy, and a prior positive COVID-19 test result were all significantly associated with positive vaccine acceptance. It is perhaps not surprising that respondents with a comorbidity and those who were pregnant were more likely to accept a COVID-19 vaccine since both having certain medical conditions (including diabetes, lung and heart conditions) and being pregnant or recently pregnant are associated with a higher risk of severe COVID-19 outcomes including hospitalization, ventilation, and death [53]. However, our finding that pregnant women (72%) were more likely to accept the vaccine than postpartum women (57%) contrasts with results from Skjefte et al. [27], in which 52% of pregnant women and 73% of non-pregnant mothers intended to receive the vaccine. In one meta-analysis, more breastfeeding women were willing to receive a COVID-19 vaccine than pregnant women (62% vs. 49%, respectively) [21], while in another meta-analysis vaccine acceptance among pregnant and breastfeeding women was roughly similar (51% vs. 47%, respectively) [23].

Those with a previous positive COVID-19 test may also have been more willing to get a vaccine themselves and for their families after experiencing the disease. Our findings contrast with one meta-analysis, in which presence of a comorbidity or a history of COVID-19 infection was not significantly associated with intent to receive a COVID-19 vaccination, although uptake of other vaccines such as influenza and/or Tdap during pregnancy was associated with increased COVID-19 vaccine acceptance [20]. In our study, age was not associated with vaccine acceptance, consistent with results from two meta-analyses [20,22]. However, older age was associated with higher vaccine acceptance in pregnant women in two systematic reviews, one based on worldwide studies and one based on US studies [10,28].

In our survey, across all four countries, the most common reason cited for vaccine hesitancy was “safety/side effect concerns” (51%). It was also the most common reason cited in each of the four countries. Safety and side effect concerns were also the most common reasons for COVID-19 vaccine hesitancy in many other surveys of pregnant women [28], consistent with findings from studies about the use other vaccines during pregnancy [54]. For example, Skjefte et al. found that 66% of pregnant women who expressed COVID-19 vaccine hesitancy did so due to concern over side effects to the developing baby and 49% wanted additional safety and effectiveness data among pregnant women [27].

One strategy to improve vaccine hesitancy among pregnant women is to improve healthcare provider outreach and recommendations to vaccinate. A healthcare provider recommendation strongly influences a pregnant woman’s decision to vaccinate [28,54,55,56], and physician hesitancy to recommend COVID-19 vaccination for pregnant patients is a contributor to low uptake in this population [56]. Efforts should include strategies to both improve hesitancy among physicians as well as more strongly communicate the importance of receiving the vaccine (and the benefits to both mother and fetus) to pregnant women. For women without access to routine healthcare, using social media or local champions (e.g., community health workers) who are trusted in their communities, along with public education, may be a strategy to improve vaccine hesitancy.

Another strategy to improve vaccine hesitancy going forward is to include pregnant women in vaccine clinical trials, from which they have been historically excluded due to concerns about the safety of the fetus. In our study and in others [28], concerns about safety and side effects are highlighted as the main reasons for hesitancy among pregnant women, and thus generating reassuring safety data earlier could improve vaccine acceptance.

Strengths of our study are that the survey was large and included a global sample, allowing us to obtain diverse perspectives on vaccine hesitancy among pregnant and postpartum women and make intercountry comparisons. In addition, our survey collected information not just about participants’ views on receiving a vaccine themselves, but also their views on vaccinations for their children/other family members.

One limitation of our study is that some important elements (i.e., socioeconomic status and educational level) that can be confounding factors in vaccine acceptability were not included. Secondly, information about acceptance of other non-COVID-19 vaccines recommended during pregnancy or knowing someone who died from COVID-19 was not obtained. In addition, there was likely some selection bias because the survey was conducted online; people with higher education levels and greater access to technology were more likely than the general population to participate. Finally, responses may not be generalizable to all regions and acceptability may be different depending on factors such as COVID-19 incidence in the various countries.

Vaccine acceptability and hesitancy are multifactorial and can change over time and therefore vaccine willingness may be different now than when our survey was conducted. For example, as data continue to accumulate regarding primary vaccine and booster safety and effectiveness during pregnancy and breastfeeding, attitudes may continue to evolve, just as guidelines and public health recommendations have evolved. In addition, we asked women about taking a COVID-19 vaccine *during* pregnancy, a question that is less relevant now because pregnancy is a time limited condition, and as more and more women receive the vaccine, a greater number have already been vaccinated by the time they become pregnant and vaccination rates during pregnancy have decreased [45]. For example, after an initial uptick in COVID-19 vaccination during pregnancy in the first half of 2021 in the US, rates began to fall [45]; among the 71% of pregnant women who had been vaccinated by 29 October 2022, 99% had received the vaccine prior to pregnancy [45].

## 5. Conclusions

Pregnant and lactating women were not originally included in the development programs of COVID-19 vaccines and so controlled data are not available. However, observational data on safety and effectiveness from real world use of the vaccine in this population are accumulating and will help inform decision making about vaccination by pregnant women and their health care providers [9,10,11]. Findings from this study provide important information that can be used to understand attitudes and concerns of the pregnant and postpartum populations in a snapshot in 2020. Reducing complications due to COVID-19 requires targeted outreach to address concerns of pregnant and postpartum women with low vaccine acceptance levels while data on safety and efficacy continue to accumulate. Effective strategies to disseminate evidence that demonstrates vaccine safety and efficacy during pregnancy can facilitate informed decisions for women of childbearing potential, pregnant women and their providers to reduce the risk of COVID-19 during pregnancy.

## Figures and Tables

**Table 1:** Baseline demographic characteristics.

	Total (N = 2010)	United States (N = 500)	United Kingdom (N = 500)	India (N = 510)	Brazil (N = 500)
	N (%)				
Age (years)					
18–29	703 (35)	226 (45)	166 (33)	90 (18)	221 (44)
30–39	1103 (55)	211 (42)	276 (55)	381 (75)	235 (47)
40–49	180 (9)	49 (10)	54 (11)	37 (7)	40 (8)
50–59	24 (1)	14 (3)	4 (1)	2 (0)	4 (1)
Stage					
1st trimester	366 (18)	76 (15)	61 (12)	149 (29)	80 (16)
2nd trimester	649 (32)	162 (32)	146 (29)	213 (42)	128 (26)
3rd trimester	375 (19)	112 (22)	92 (18)	99 (19)	72 (14)
Postpartum	620 (31)	150 (30)	201 (40)	49 (10)	220 (44)
COVID-19 test ^†^					
Ever positive	357 (28)	89 (30)	95 (35)	137 (30)	36 (15)
Not positive	900 (72)	205 (70)	174 (65)	320 (70)	201 (85)
Comorbidity					
Any	934 (46)	221 (44)	224 (45)	376 (74)	113 (23)
None	1076 (54)	279 (56)	276 (55)	134 (26)	387 (77)

^†^ N = 1257.

## Data Availability

The data presented in this study are available in Supplementary Spreadsheet S1.

## Supplementary Materials

The following supporting information can be downloaded at: https://www.mdpi.com/article/10.3390/vaccines11020390/s1, Table S1: Univariate analyses by country and across countries for potential prognostic factors for openness to receiving a COVID-19 vaccine oneself (vs. Neutral/Unsure and Not Open) among pregnant and postpartum women; Table S2: Univariate analyses by country and across countries for potential prognostic factors for openness to children/other family members taking receiving a COVID-19 vaccine (vs. Neutral/Unsure and Not Open) among pregnant and postpartum women; Table S3: Multivariate analyses by country and across countries for potential prognostic factors for openness to receiving a COVID-19 vaccine oneself (vs. Neutral/Unsure and Not Open) among pregnant and postpartum women; Table S4: Multivariate analyses by country and across countries for potential prognostic factors for openness to children/other family members receiving a COVID-19 vaccine (vs. Neutral/Unsure and Not Open) among pregnant and postpartum women; Spreadsheet S1: Raw data.
