# Supplementary material for: Global Cross-Sectional Study Evaluating the Attitudes towards a COVID-19 Vaccine in Pregnant and Postpartum Women
Source: Vaccines (Basel). 2023 Feb 8;11(2):390. doi: 10.3390/vaccines11020390 (PMC9961540; doi:10.3390/vaccines11020390)
Supplement: Supplementary file 1 [file vaccines-11-00390-s001.zip › Supplementary Tables S1-S4_30Jan22.pdf]

Table S1. Univariate analyses by country and across countries for potential prognostic factors for openness to receiving a COVID-19 vaccine oneself (vs. Neutral/Unsure and Not Open) among pregnant and postpartum women. Odds ratios greater than one indicate that the respondents in the comparison group were more open than the reference group to vaccine acceptability.

Prognostic Factor	United States		United Kingdom		India		Brazil		Across All Countries	
	Odds Ratio	95% CI	Odds Ratio	95% CI	Odds Ratio	95% CI	Odds Ratio	95% CI	Odds Ratio	95% CI
**Any Comorbidity vs. No Comorbidity**	2.25	1.57, 3.22	2.08	1.44, 3.00	8.55	4.85, 15.08	1.16	0.72, 1.85	2.61	2.14, 3.18
**COVID-19 Positive vs. Not Positive**	3.47	2.07, 5.84	6.95	3.60, 13.42	4.19	1.77, 9.93	1.45	0.65, 3.26	3.84	2.78, 5.30
**Pregnant vs. Postpartum**	1.83	1.24, 2.69	1.67	1.16, 2.41	1.85	0.88, 3.92	1.46	0.99, 2.16	1.97	1.62, 2.40
**Age 18 – 29 vs. 40+**	0.83	0.47, 1.45	1.24	0.68, 2.26	0.40	0.13, 1.26	0.68	0.32, 1.45	0.85	0.61, 1.17
**Age 30 – 39 vs. 40+**	0.86	0.49, 1.51	1.73	0.98, 3.06	0.92	0.31, 2.73	0.74	0.35, 1.58	1.35	0.99, 1.86

CI, Confidence interval

Table S2. Univariate analyses by country and across countries for potential prognostic factors for openness to children/other family members receiving a COVID-19 vaccine (vs. Neutral/Unsure and Not Open) among pregnant and postpartum women. Odds ratios greater than one indicate that the respondents in the comparison group were more open than the reference group to vaccine acceptability.

Prognostic Factor	United States		United Kingdom		India		Brazil		Across All Countries	
	Odds Ratio	95% CI	Odds Ratio	95% CI	Odds Ratio	95% CI	Odds Ratio	95% CI	Odds Ratio	95% CI
**Any Comorbidity vs. No Comorbidity**	1.94	1.36, 2.78	2.09	1.44, 3.03	6.63	3.85, 11.43	0.80	0.51, 1.26	2.24	1.84, 2.71
**COVID-19 Positive vs. Not Positive**	2.91	1.79, 4.74	7.88	3.86, 16.09	9.43	2.91, 30.53	0.89	0.42, 1.85	3.54	2.59, 4.84
**Pregnant vs. Postpartum**	2.20	1.48, 3.28	1.51	1.05, 2.18	2.05	0.99, 4.24	1.49	1.07, 2.20	1.97	1.62, 2.40
**Age 18 – 29 vs. 40+**	0.59	0.33, 1.03	1.14	0.62, 2.07	0.30	0.10, 0.92	0.88	0.43, 1.82	0.74	0.54, 1.03
**Age 30 – 39 vs. 40+**	0.70	0.40, 1.24	1.71	0.97, 3.04	1.00	0.34, 2.97	1.02	0.50, 2.11	1.37	0.99, 1.89

CI, Confidence interval

Table S3. Multivariate analyses by country and across countries for potential prognostic factors for openness to receiving a COVID-19 vaccine oneself (vs. Neutral/Unsure and Not Open) among pregnant and postpartum women. Odds ratios greater than one indicate that the respondents in the comparison group were more open than the reference group to vaccine acceptability.

Prognostic Factor	United States		United Kingdom		India		Brazil		Across All Countries	
	Odds Ratio	95% CI	Odds Ratio	95% CI	Odds Ratio	95% CI	Odds Ratio	95% CI	Odds Ratio	95% CI
**Any Comorbidity vs. No Comorbidity**	1.73	1.16, 2.56	1.33	0.88, 2.01	7.19	3.76, 13.75	1.05	0.64, 1.72	1.85	1.49, 2.29
**COVID-19 Positive vs. Not Positive**	2.47	1.38, 4.41	5.42	2.68, 10.95	1.51	0.58, 3.96	1.33	0.57, 3.08	2.49	1.76, 3.52
**Pregnant vs. Postpartum**	1.54	1.03, 2.30	1.23	0.83, 1.82	1.02	0.45, 2.33	1.41	0.94, 2.10	1.51	1.23, 1.86
**Age 18 – 29 vs. 40+**	1.24	0.67, 2.27	1.01	0.59, 2.05	0.73	0.21, 2.53	0.75	0.35, 1.63	1.08	0.77, 1.52
**Age 30 – 39 vs. 40+**	1.20	0.66, 2.20	1.60	0.88, 2.91	0.87	0.27, 2.78	0.78	0.36, 1.67	1.48	1.06, 2.06

CI, Confidence interval

Table S4. Multivariate analyses by country and across countries for potential prognostic factors for openness to children/other family members receiving a COVID-19 vaccine (vs. Neutral/Unsure and Not Open) among pregnant and postpartum women. Odds ratios greater than one indicate that the respondents in the comparison group were more open than the reference group to vaccine acceptability.

Prognostic Factor	United States		United Kingdom		India		Brazil		Across All Countries	
	Odds Ratio	95% CI	Odds Ratio	95% CI	Odds Ratio	95% CI	Odds Ratio	95% CI	Odds Ratio	95% CI
**Any Comorbidity vs. No Comorbidity**	1.51	1.02, 2.24	1.36	0.89, 2.06	4.00	2.21, 7.22	0.76	0.47, 1.22	1.55	1.25, 1.91
**COVID-19 Positive vs. Not Positive**	1.94	1.12, 3.36	6.33	3.00, 13.50	4.50	1.32, 15.32	0.93	0.43, 2.01	2.47	1.76, 3.46
**Pregnant vs. Postpartum**	1.91	1.27, 2.88	1.09	0.74, 1.63	1.13	0.50, 2.55	1.51	1.01, 2.26	1.54	1.25, 1.89
**Age 18 – 29 vs. 40+**	0.79	0.44, 1.43	0.99	0.53, 1.85	0.54	0.16, 1.81	0.97	0.46, 2.02	0.92	0.66, 1.30
**Age 30 – 39 vs. 40+**	0.92	0.50, 1.66	1.56	0.86, 2.84	1.06	0.33, 3.35	1.08	0.52, 2.23	1.50	1.08, 2.08

CI, Confidence interval
